# Migrations of young spotted seals (*Phoca largha*) from Peter the Great Bay, Sea of Japan/East Sea, and the pattern of their use of seasonal habitats

**DOI:** 10.1371/journal.pone.0244232

**Published:** 2021-01-05

**Authors:** Alexey M. Trukhin, Peter A. Permyakov, Sergey D. Ryazanov, Vyacheslav B. Lobanov, Hyun Woo Kim, Young Min Choi, Hawsun Sohn

**Affiliations:** 1 Far Eastern Branch of the Russian Academy of Science, V.I. Il’ichev Pacific Oceanological Institute, Vladivostok, Russia; 2 National Institute of Fisheries Science, Cetacean Research Institute, Ulsan, Republic of Korea; University of Waikato, NEW ZEALAND

## Abstract

We studied the migrations of young spotted seals during their annual cycle. In May 2017, we attached satellite tags (SPOT-293A) to three individuals (two underyearlings and one yearling) captured at their breeding ground in Peter the Great Bay, western Sea of Japan/East Sea. The operational time of the installed tags ranged from 207 to 333 days; a total of 27195 locations were uploaded. All three seals migrated east and further north along the coast of the mainland. The average daily migration speed of the seals ranged between 70 and 135 km/day. The yearling moved faster than the underyearlings. During early August, they arrived at their summer habitats, which were located in the northern part of the Tatar Strait (Sea of Japan/East Sea) for the underyearling seals and in Aniva Bay (Sea of Okhotsk) for the yearling seal. While moving from the place of tagging to the summer feeding grounds, the seals covered a distance of 2300 to 3100 km. From August to October, each seal permanently stayed within the same isolated area. The reverse migration of all three seals began in November. When the seals traveled south, they used the same routes by which they had moved north in the spring, but they moved at a faster speed. By December, two seals returned to their natal islands, where both stayed until their transmitters stopped sending signals (in March 2018).

## Introduction

The spotted seal (*Phoca largha*), sometimes referred to as the largha seal, is a pinniped species inhabiting the northern Pacific Ocean and the adjacent Arctic seas [1]. Spotted seals are most common in the Sea of Okhotsk and the Bering Sea, where up to 95% of the world's spotted seal population is concentrated, and the distribution of seals during the year is characterized by pronounced interseasonal variations [2, 3]. During the breeding season in the Sea of Okhotsk and the Bering Sea, spotted seals aggregate on pack ice, mainly in the shelf-edge zone. When these seas become free of ice, the seals haul-out on the shores of the mainland and islands, where they spend a significant part of the open-water period of their annual cycle. Data on the migration of spotted seals became available only with broad application of tagging. Tracking of tagged animals showed that spotted seals make extensive seasonal migrations to various parts of their range [4–9].

Within the species range, spotted seals form eight reproductive concentrations, one of which is located in Peter the Great Bay (hereafter PGB), the western Sea of Japan/East Sea, at the southern boundary of the range [10]. The PGB habitat of the spotted seals has been known since the first half of the 20th century [11–13], but there were few studies of the seals in this habitat before the early 1960s. Spotted seals from PGB have distinctive morphological, biological, and ecological features: larger body size, grayish-colored lanugo, earlier period of reproduction, and other features. These features distinguish them from seals inhabiting other areas of the range, and, for this reason, the group of seals from PGB was assigned the status of “independent population” [14].

The habitat conditions of spotted seals in PGB are associated with a number of serious anthropogenic threats (disturbance, active shipping, and commercial fishing, tourism). Nevertheless the population is currently growing [15, 16], mainly because the breeding grounds of seals in the Bay are confined to the protected water area of the Far Eastern Marine Biosphere Reserve.

After completion of the breeding season, the number of spotted seals in PGB is reduced, which suggests the departure of a significant part of the local population from the Bay to the north and south [17]. The first reliable data on migrations of underyearling spotted seals born in PGB were obtained after marking pups with plastic tags. Recapture of tagged seals indicated that they are able to travel at least 1400 km away from PBG after weaning [6, 18].

Visual observations of tagged seals and the findings of dead spotted seals with tags made it evident that spotted seals from PGB reach the northern Sea of Japan/East Sea, the southern Sea of Okhotsk, and waters off the Pacific coast of Hokkaido [6, 18]. Re-sightings of hot-branded spotted seals also reveal the possibility of southward migrations from PGB [19]. At the same time, more specific questions about spotted seals' migrations from PGB remain unanswered. In May 2017, we tagged three individuals in PGB with satellite transmitters to study their migration. For the study, the following objectives were set up: to determine the direction and distance of seals' migration to and from their summer–autumn feeding grounds; to locate areas of their stay during the feeding period (in summer and autumn haul-outs); and to clarify the locations and sizes of the summer, autumn, and winter habitats used by the animals.

## Materials and methods

### Ethics statement

Animal handling and tag attachment procedures were permitted by the Federal Agency for Fisheries of the Ministry of Agriculture of the Russian Federation and approved by the Pacific Fisheries Research Center. All applicable international, national, and institutional principles for the care and use of animals were followed.

### Capturing and tagging seals

Three juvenile spotted seals were tagged on May 18 and 19, 2017. ARGOS satellite tags SPOT-293A (ARGOS SPOT, Wildlife Computers, Inc., Redmond, WA, USA) were attached to the top of the head of each seal using Loctite™ Type 422 glue (Henkel Corp., Düsseldorf, Germany). The tags regularly uploaded data about their location through the ARGOS satellite network using the Doppler effect. Information from wet/dry sensors was reported hourly as the percentage of time when the tags were switched into a "dry" mode. All tagged seals were captured in the Rimsky-Korsakov Archipelago located in the central part of PGB (42°38ʹ–42°34ʹ N, 131°21ʹ–131°31ʹ E). The seals were captured at their coastal haul-out sites using a hoop net. Before tagging, the sex of each captured individual was identified and its body weight, length, and girth were measured. The age of each seal was determined by such characteristics as body length and size of claws. The tagged seals were a male and a female aged 2.5 to 3 months (hereafter referred to as the underyearling male and the underyearling female) and a yearling male.

### Data processing

The data were filtered using ARGOS (Kalman filtering algorithm) and additionally processed by the SDA-filtering algorithm [20] scripted in the “argosfilter” package available in R [21].

The critical values for speed were set at 3.8 m/s at distances greater than 5 km [22] and at 10 m/s irrespective of distance; the angle between two spans of track could not be greater than 15° at distances from 2.5 to 5.0 km and 25° at distances >5.0 km. All locations >1 km inland from the coast after applying the SDA filter were removed from the dataset. All data were normal (Shapiro-Wilk normality test, S1 File) and subsequently analysis of variance (One-way ANOVA, S2 File) and t-tests (Unpaired two-tailed pairwise t-tests, S3 File) were used for further analyses. The geographic boundaries of statistically significant (*p* < 0.05) habitat areas for seals were drawn using a hotspot analysis (Getis-Ord Gi*, Spatial Statistics, ArcMap). During this procedure, a certain large polygon (space of the Sea of Japan/East Sea in our case) was divided into a “fishnet” grid of square cells with a side length of 15 km. Registrations of individual seals were aggregated inside the neighborhood of each cell, i.e., the cell itself and the cells adjoining it. The density of registrations of individual seals in the neighborhood was then compared with the density of registrations of individual seals in the large polygon, and the Gi* (read: *Gi-asterisk*) statistics with corresponding Gi*-bins (levels of significance) were calculated. The procedure was run for each cell in the large polygon. The results indicated the areas where cells with either high or low densities of registrations were clustered spatially. The areas of “hot” cells (high density, Gi*-bins ≥ 2) were considered significant habitat areas for the spotted seals under study (hereafter, key areas). Attaching a 10-km buffer zone to each cell made it possible to outline these areas in a relatively uninterrupted manner.

Delineation of areas with statistically significant increases or reductions in horizontal speeds was also performed through hotspot analysis, but only in the form of point neighborhood, i.e., as an aggregation of location itself and the number of locations closest to it. In the current research, we considered the distances passed by seals per day (hereinafter, daily speeds). Denotation of points as “hot” (high speeds, Gi*-bins ≥ 2) or “cold” (low speeds, Gi*-bins ≤ – 2) meant a significantly (*p* < 0.05) increased or decreased horizontal speed of a certain seal at the given coordinates. We assumed that a significant decrease in horizontal speed close to land in combination with tags switching into a "dry" mode indicated the presence of a coastal haul-out of seals at the given coordinates; clusters of “cold” spots in offshore were assumed to be feeding sites.

Data were processed by regular statistical procedures [23]; ArcMap 10.3, R3.4.3, and GraphPad Prism 6 software were used in the analysis.

## Results

In total, all three installed satellite tags uploaded data about 27195 locations (8867 locations remained after filtering); the service time of the tags ranged between 207 and 333 days (Table 1).

**Table 1:** Biometrics of the tagged spotted seals and sizes of datasets received from the satellite tags.

	Satellite tag serial ID		
Variable	94842	148660	148661
Sex	Female	Male	Male
Age	2.5–3 months	1 year	2.5–3 months
Body weight, kg	28.4	35.6	32.2
Body length, cm	116.5	123.5	111.0
Date of tagging	May 18, 2017	May 18, 2017	May 19, 2017
Date of last upload	March 30, 2018	December 11, 2017	April 17, 2018
Total duration of tag activity, days	316	207	333
Number of locations before filtering	8805	7803	10587
Number of locations after filtering	3131	2257	3479

### Direction and speed of migration

All three tagged seals started moving from their natal islands within the first 5 days after the tagging procedure. Two seals (both males, #148660 and #148661) headed east; the underyearling female (#94842) traveled south along the coast up to the Korean Peninsula, about 280 km from PGB. However, the female seal returned to PGB by June 4 and, like both male seals before her, proceeded east and then north along the coast of the Russian mainland. By June 10, all three seals were out of PGB; one of them (yearling male #148661) was already in the Tatar Strait on that date.

**Fig 1:**
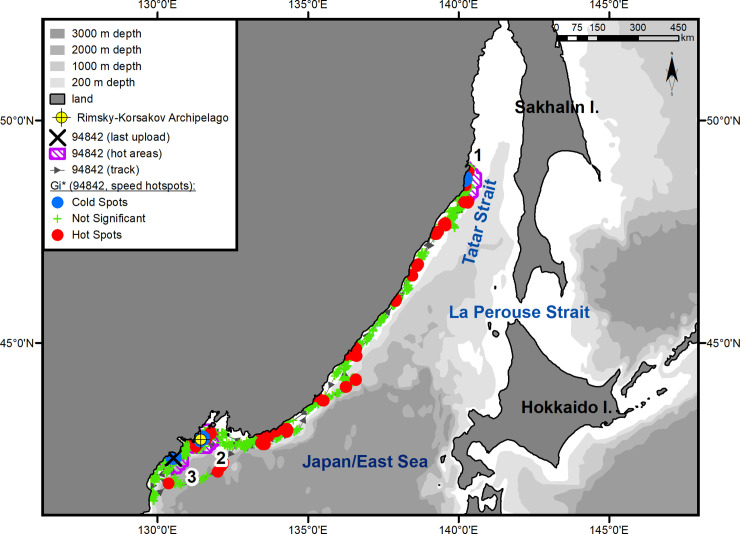
Summer–autumn migrations of underyearling female #94842.

**Fig 2:**
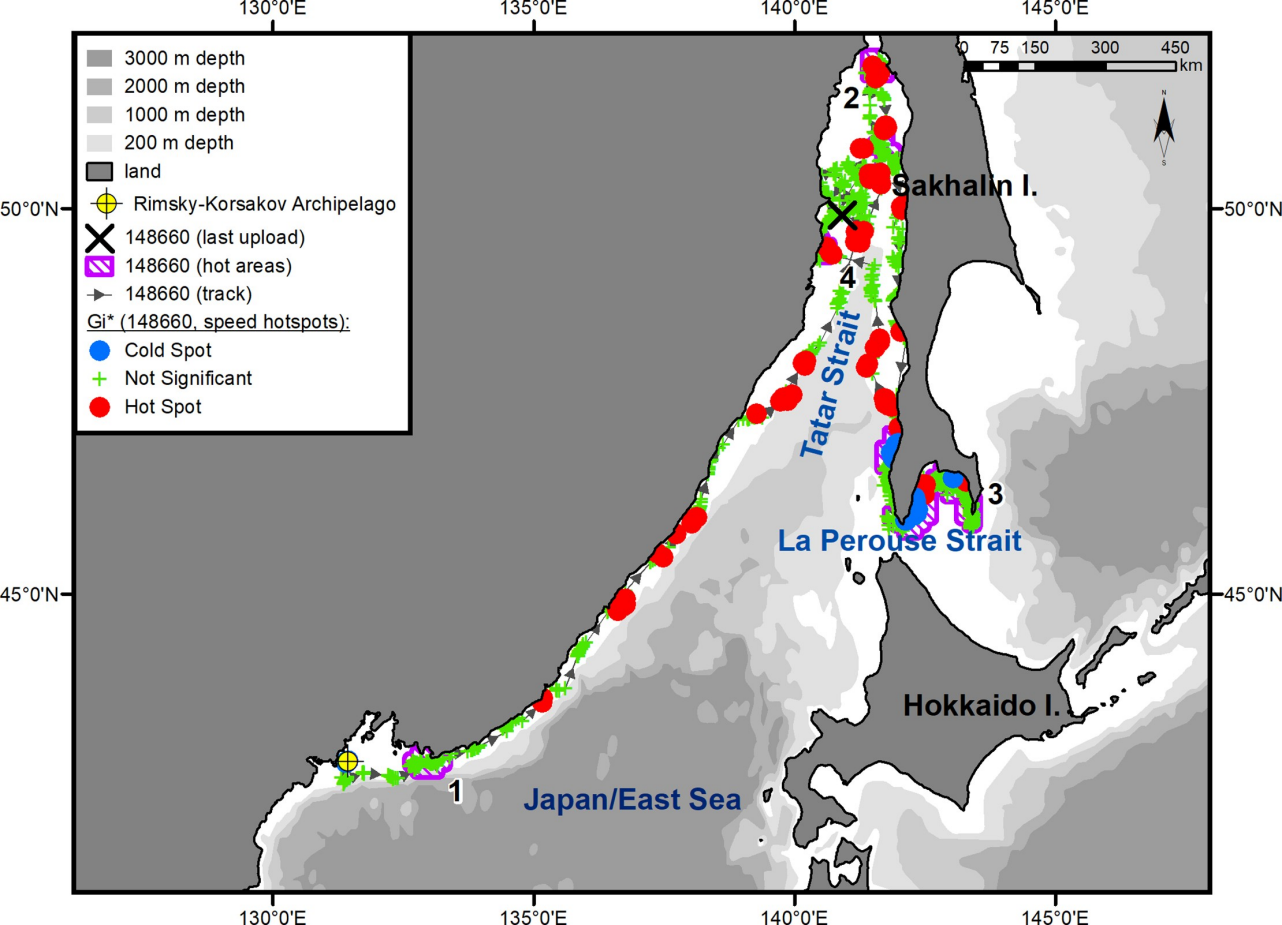
Summer–autumn migrations of yearling male #148660.

**Fig 3:**
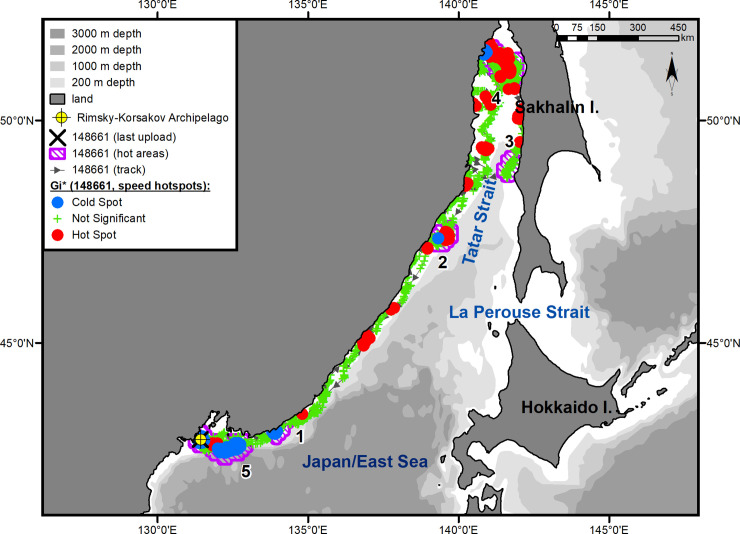
Summer–autumn migrations of underyearling male #148661.

The seals moved along the coast of the mainland over the continental slope, without going beyond the 200-m isobath on most of their route (Figs 1–3). Two- to five-day-long episodes of traveling over the 1-km isobath were typical only for the underyearling female (Fig 1). The yearling male proceeded farther north than the others, reached Tyk Bay (the western coast of Sakhalin Island, 51°47ʹ N, 141°44ʹ E) by the end of the second 10 days of July, then turned to the south, and, keeping to the coastal waters of western Sakhalin, entered Aniva Bay through the La Perouse Strait (Fig 3).

Short (up to 1 day), statistically nonsignificant (hotspot analysis,– 2 > Gi*-bins < 2) reductions in daily speeds accompanied by switching of tags into a "dry" mode were observed for all three seals during their migration.

The seals significantly differed from one another in their daily speed of migration between key areas (ANOVA, *F*_(4, 152)_ = 16.06, *p* < 0.001) (One-way ANOVA, S2 File). In pairwise tests, there were no differences between the two underyearling seals (#94842 and #148661; *t*-test, *p* = 0.78) (Unpaired two-tailed pairwise t-tests, S3 File), but the differences between the underyearling seals and the yearling male were significant (*p* < 0.001 for both comparisons). The average daily speed was 72 km/day (95% Confidence Interval (CI) = 61–82 km/day) for the underyearling female and 70 km/day (CI = 61–78 km/day) for the underyearling male. The daily speed of the yearling male during his migration to the feeding grounds along the coast of the Asian mainland was almost twice as high as that for the underyearling seals: 135 km/day (CI = 117–155 km/day) (Fig 2). The yearling male's migration from Tyk Bay to Aniva Bay occurred at an average speed of 124 km/day (CI = 75–173 km/day). From the tagging site to Aniva Bay, the yearling male covered a distance of more than 3100 km (up to 4600 km, taking into account roams in “hot” areas) (Fig 2). The routes to the summer and autumn habitats were 3000 km (up to 4100 km, taking into account roams in “hot” areas) for the underyearling male (Fig 3) and 2300 km (up to 3600 km, taking into account the southwestern trip; on the route to the summer feeding ground, no “hot” areas were identified) for the underyearling female (Fig 1).

In November, the seals began migrating back to their natal sites. Both underyearling seals moved in the southern direction along the Asian coast (Figs 1 and 3). The yearling male, after leaving Aniva Bay, went through the La Perouse Strait into the Sea of Japan/East Sea and began moving north along the western coast of Sakhalin Island. After he reached the northern Tatar Strait, his tag stopped sending signals (Fig 2). A characteristic feature of the seals' reverse migration was a higher swimming speed (*t*-test, *p* < 0.01) (Unpaired two-tailed pairwise t-tests, S3 File) and a shorter distance covered: on reverse migration, the average daily speed of the underyearling female was 141 km/day (CI = 96–185 km/day), and the distance covered was 1700 km (compared with 2300 km during the summer migration); the migration speed of the underyearling male was 113 km/day (CI = 85–142 km/day), and the distance was 1800 km (compared with 3000 km during the summer migration). The initial, tracked part of the yearling male's route of reverse migration was 500 km, which the seal covered at an average speed of 185 km/day (CI = 163–205 km/day).

### Pattern of use of the water area during the summer–autumn feeding period

From August to October, seal kept within a well-defined key areas; during this period, all spatial movements of seals occurred locally.

#### Underyearling female #94842

**Fig 4:**
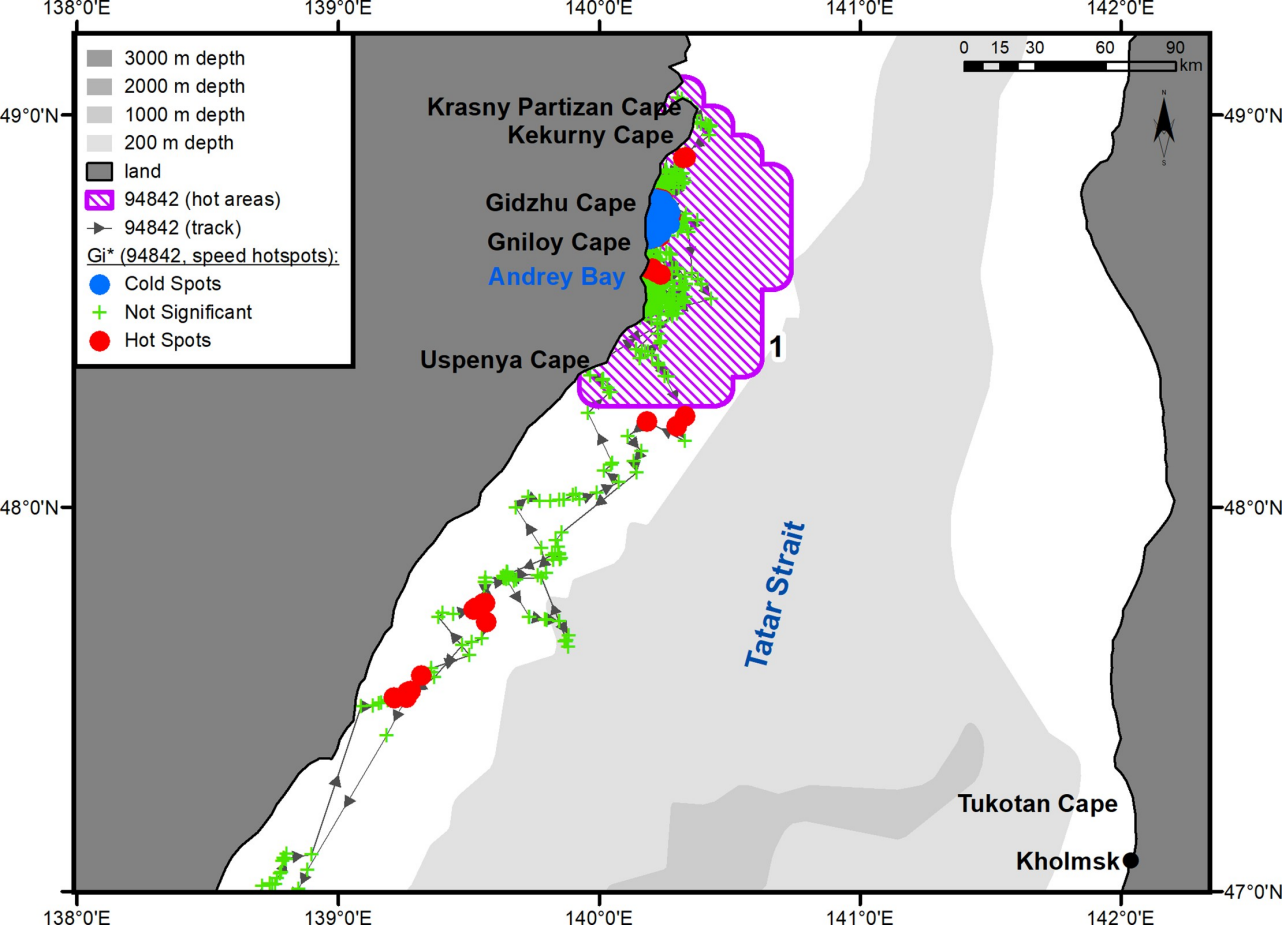
Summer–autumn habitat area of underyearling female #94842. The numbers of the hot areas are the same as in Fig 1.

Immediately after being tagged, the underyearling female moved southwest and reached Eorang Pt., coast of Korean Peninsula (41°22' N, 129°48' E). Her northeastward migration began in early June (Fig 1). The movement to the summer feeding ground was completed on July 14. In the summer, the female was feeding within a quite limited key area bounded by Peschany Cape (48°26' N, 140°10' E) on the south and Krasny Partizan Cape (48°58' N, 140°23' E) on the north (Fig 1, hot area 1). From the time of arrival in the feeding area (July 14), the seal did not go farther than 30 km away from the shore and tended to remain in Andrey Bay (estuaries of the Kopka and Koppi rivers) (48°33' N, 140°10' E) and the segment of coast between Gniloi Cape (48°38' N, 140°11' E) and Kekurny Cape (48°55' N, 140°21' E) (Fig 4). Between Gniloi Cape and Gidzhu Cape (in the Gidzhu River estuary) (48°44' N, 140°11' E), a cluster of “cold” points outlined an area with a significant reduction in the seal's horizontal speed (hotspot analysis, Gi*-bins ≤ – 2). The tag regularly switched into a "dry" mode while in this area.

#### Yearling male #148660

**Fig 5:**
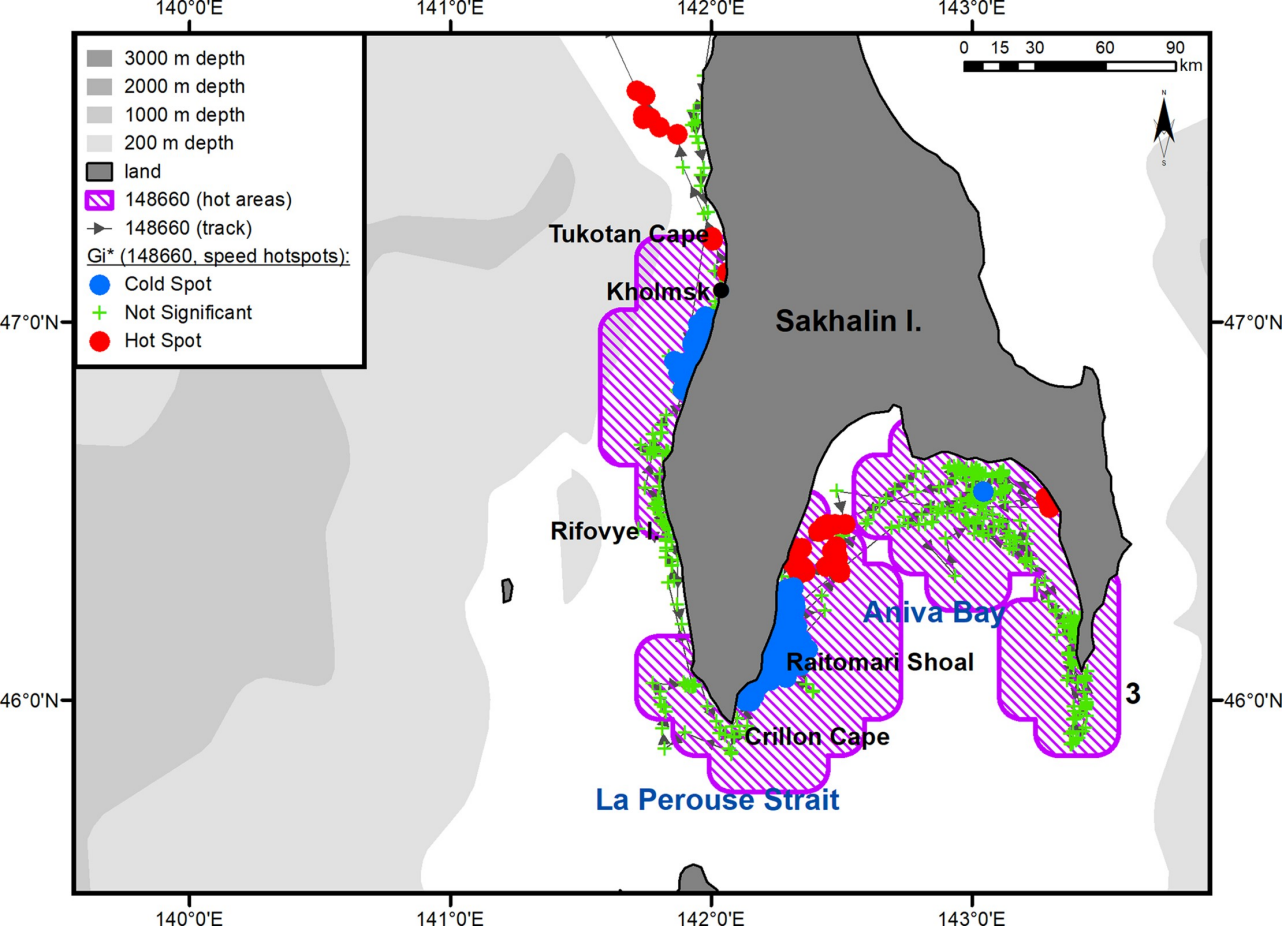
Summer–autumn feeding area of yearling male #148660. The numbers of the hot areas are the same as in Fig 2.

On the way to the summer feeding ground, the yearling male made short stopovers in two limited key areas, of which one was located in the eastern part of PGB (Fig 2, hot area 1) and the other was in the northern Tatar Strait (Fig 2, hot area 2). In the summer, the male was feeding in Aniva Bay, where he arrived on July 14 (Fig 2, hot area 3). After going around Crillon Cape, the seal came first to the Raitomari Shoal (46°04' N, 142°12' E). The most important ground for this individual, where he spent almost 60% of his feeding time (83 out of 131 days) (Fig 5), was located north of the Shoal up to the estuaries of the Kura (46°14' N, 142°14' E) and Ulyanovka (46°16' N, 142°14' E) rivers. This area was characterized by a statistically significant reduction in horizontal speed (hotspot analysis, Gi*-bins ≤ – 2) and a regular switch of the tag into a "dry" mode. In mid-August, the seal moved for a while to the eastern coast of Aniva Bay and remained there from August 14 to 27. During the summer–autumn feeding period, the seal made another short trip (from September 18 to 29) to Aniva Cape. After each trip across Aniva Bay, the seal returned to the area of the Raitomari Shoal and the Ulyanovka River.

#### Underyearling male #148661

**Fig 6:**
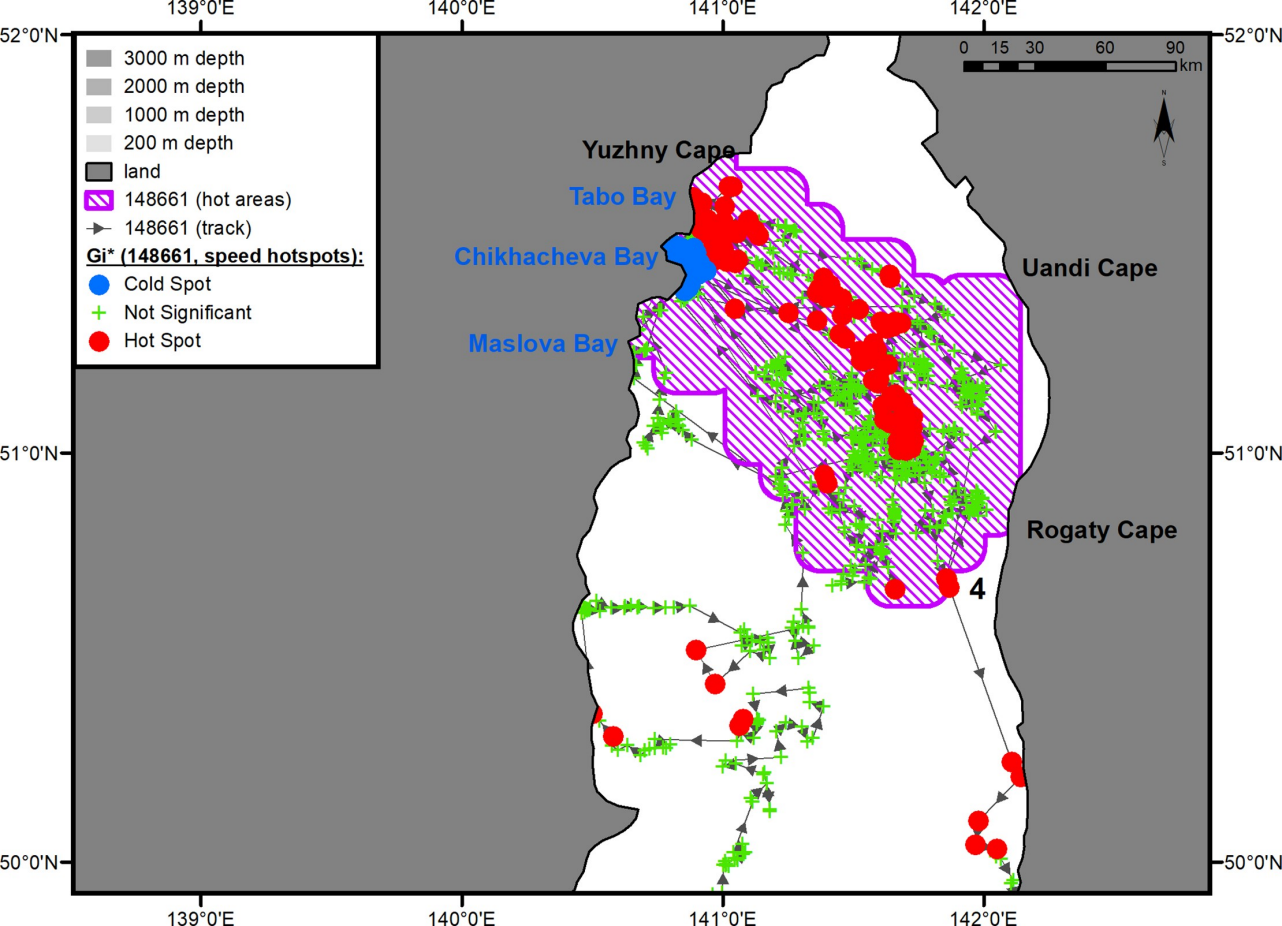
Summer–autumn feeding area (hot area 4) of underyearling male #148661. The numbers of the hot areas are the same as in Fig 3.

The juvenile male, in the same way as the yearling male, made short stopovers in small key areas located on the routes to and from the main grounds of the summer and winter habitats (Fig 3, hot areas 1–3). Shortly after tagging, on May 24, 2017, the male left PGB and entered the first such key area (Fig 3, hot area 1) extending from Kievka Bay (42°51' N, 133°38' E) to Tchernoruche Bay (43°10' N, 134°26' E). At this point, there was a significant decrease in the seal’s movement speed (hotspot analysis, Gi*-bins ≤ – 2) in the water area opposite Sokolovskaya Bay (village of Preobrazhenye). Before arriving at the main summer–autumn feeding ground, the underyearling male stayed for a long time (from June 9 to July 2, 2017) in a small key area, consisting of the sea area adjacent to the Asian coast in the west between capes Zolotoy (47°19' N, 138°59' E) and Ptichii (47°32' N, 139°06' E) and extending eastward into the Strait for almost 100 km (Fig 3, hot area 2). This individual completed his summer–autumn feeding period within the first 10 days of August in Chikhacheva Bay (51°27' N, 140°5' E) (Fig 3, hot area 3). The summer–autumn feeding area of this seal was quite extensive (14000 km^2^, excluding the 10-km buffer zone) (Fig 6). It stretched across the Tatar Strait from Maslova Bay (51°14' N, 140°40' E) to Yuzhny Cape (51°41' N, 141°06' E) on the Asian coast and from Uandi Cape (51°25' N, 142°31' E) to Rogaty Cape (50°44' N, 142°05' E) on the western coast of Sakhalin Island (Fig 3, hot area 4). The seal spent almost half of his time in Chikhacheva Bay and in the nearby Tabo Bay. The tag regularly switched into the "dry" mode while inside this area. There was a long, narrow zone of hot spots of travel speeds that extended from Tabo Bay toward Sakhalin Island. The seal made six trips from Chikhacheva Bay to the coast of Sakhalin Island and back. The duration of each trip was up to 10 days (mean = 6 days, CI ± 3 days).

### Pattern of use of the water area in the winter and early spring

#### Underyearling female #694842

**Fig 7:**
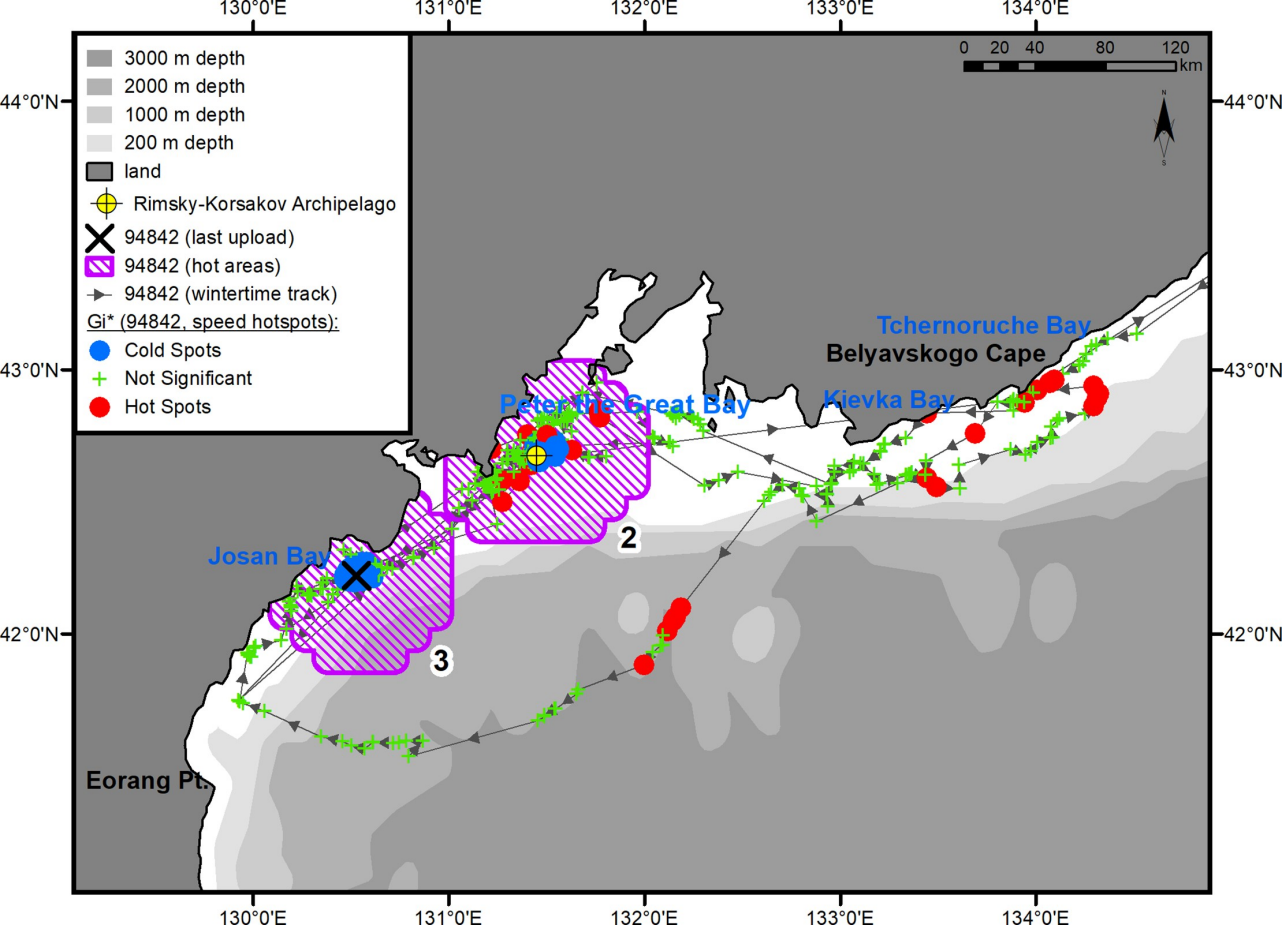
Winter habitats of underyearling female #94842. The numbers of the hot areas are the same as in Fig 1.

This seal left the feeding area on November 4 and returned to PGB after a 10-day reverse migration. From November 16 to December 4, 2017, the seal kept within the 30-km zone of the Rimsky-Korsakov Archipelago (Fig 7). In the middle of this area, a second cluster of “cold” points is outlined, overlapping the islands of the archipelago (Fig 1, hot area 2). On December 4, 2017, the female left the area and headed, first, southwest, to Josan Bay, North Korea, then northeast and reached Belyavskogo Cape. By mid-December, the seal moved back to the Korean Peninsula and entered Josan Bay. The route of this movement was far from the shore, over the 3-km isobath. The third cluster of “cold” spots (Fig 1, hot area 3) was formed in the Uam seal reserve designated as a Natural Monument No. 339 by the North Korean government [24]. Inside this area, the tag regularly entered a "dry" mode signaling the presence of a coastal haul-out. On February 10, 2018, the female entered PGB for a few days and then returned again to Josan Bay, where the last signal from her tag was received on March 30.

#### Yearling male #148660

On November 3, 2017, this male left Aniva Bay through the La Perouse Strait, went into the Sea of Japan/East Sea, and headed north along the western coast of Sakhalin Island. His movement slowed down in the segment from the Rifovye Islands (46°28' N, 141°49' E) to Tukotan Cape (47°10' N, 142°02' E) (Fig 5). South of the town of Kholmsk (between 46°47' N and 47°01' E), there was another area where the speed of the seal's horizontal movements was significantly reduced (hotspot analysis, Gi*-bins ≤ – 2). After seal crossed the Tatar Strait, its locations clustered into another key area near the Asian coast (Fig 2, hot area 4) in which, similarly to key areas 1 and 2 (Fig 2, hot areas 1 and 2), no statistically significant reduction in the seal's speed was recorded (hotspot analysis, Gi*-bins >– 2). The tag stopped signaling in the Tatar Strait on December 11.

#### Underyearling male #148661

**Fig 8:**
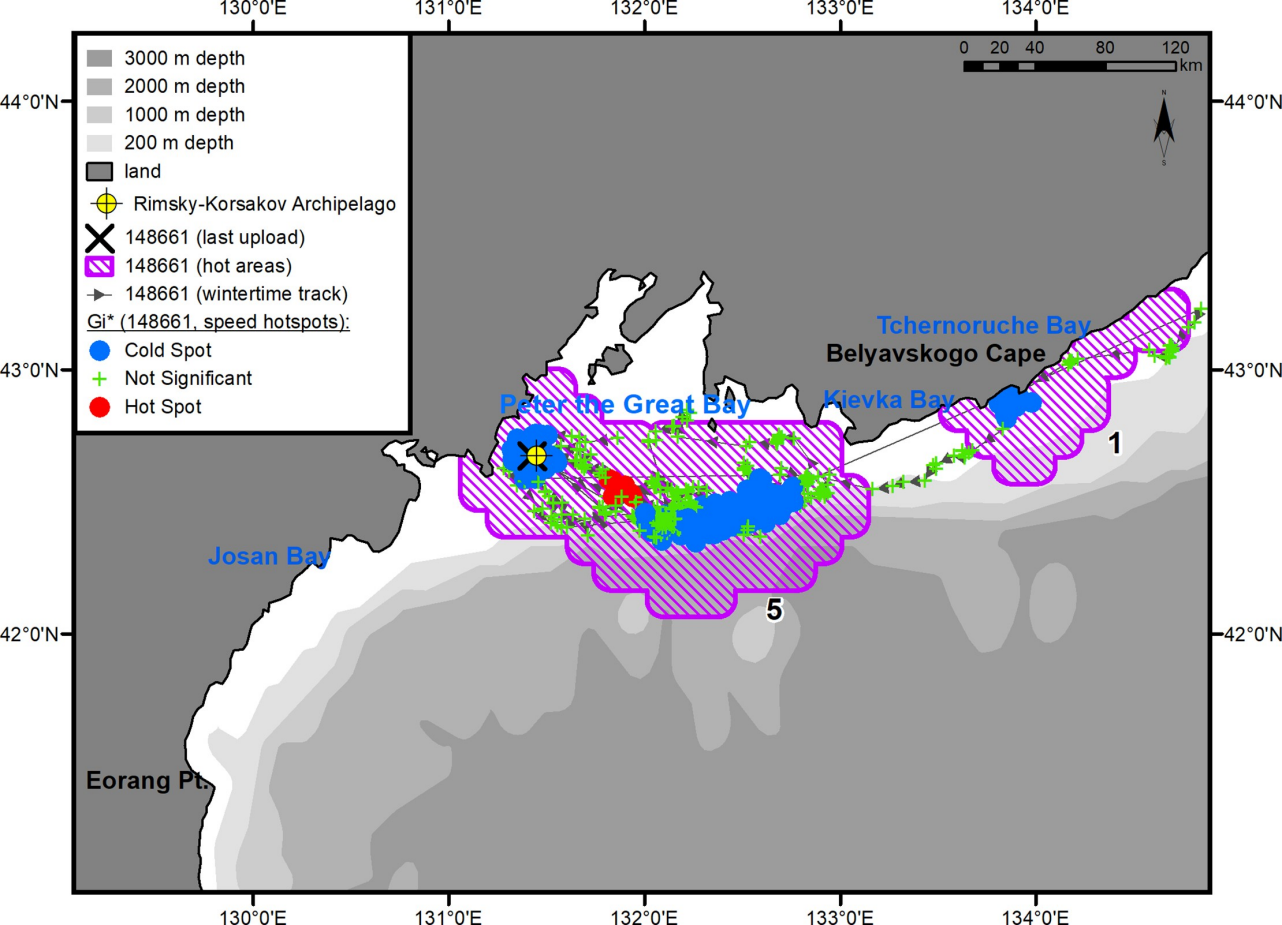
Winter habitats of underyearling male #148661. The numbers of the hot areas are the same as in Fig 3.

After completion of the summer–autumn feeding period (from August 2 to November 9, 2017), this seal moved southwest. Before the onset of reverse migration, he spent some time (from November 12 to 23, 2017) in the third key area located between capes Gavrilova (49°08' N, 142°11' E) and Lamanon (48°47' N, 141°51' E) and extending offshore for up to 80 km (Fig 3, hot area 3). The reverse migration was relatively fast, and as early as December 5, 2017, the seal arrived in the first of the additional key areas (Fig 3, hot area 1). The same as during the spring migration, the male again showed interest in Sokolovskaya Bay (42°52' N, 133°51' E), where he formed a cluster of points with significantly reduced travel speeds (hotspot analysis, Gi*-bin ≤ – 2). The tag occasionally went into a "dry" mode while in the Bay. On December 21, 2017, the seal entered PGB, where he stayed until the tag stopped sending signals (Fig 3, hot area 5). The winter habitat area was as large and complex as the feeding area of this seal (Fig 8): it occupied a total of approximately 14300 km^2^, excluding the 10-km buffer zone. Within this area, there were three smaller areas in which the seal significantly changed the speed of movements: a water area adjacent to coastal haul-out sites in the Rimsky-Korsakov Archipelago (Fig 8, rectangle *a*), a feeding area in the pelagic zone (Fig 8, rectangle *b*), and a transitional zone between the archipelago and the feeding area (Fig 8, rectangle *c*). The feeding area was located over the continental slope, southeast of PGB, and occupied approximately 1200 km^2^. During the winter–spring period of 2017–2018, the underyearling male made a total of five feeding trips lasting up to 22 days each (mean 15 days, CI ± 8 days). The return from the last feeding trip was recorded on April 1, 2018, 16 days before the last signal was received from the tag.

## Discussion

**Fig 9:**
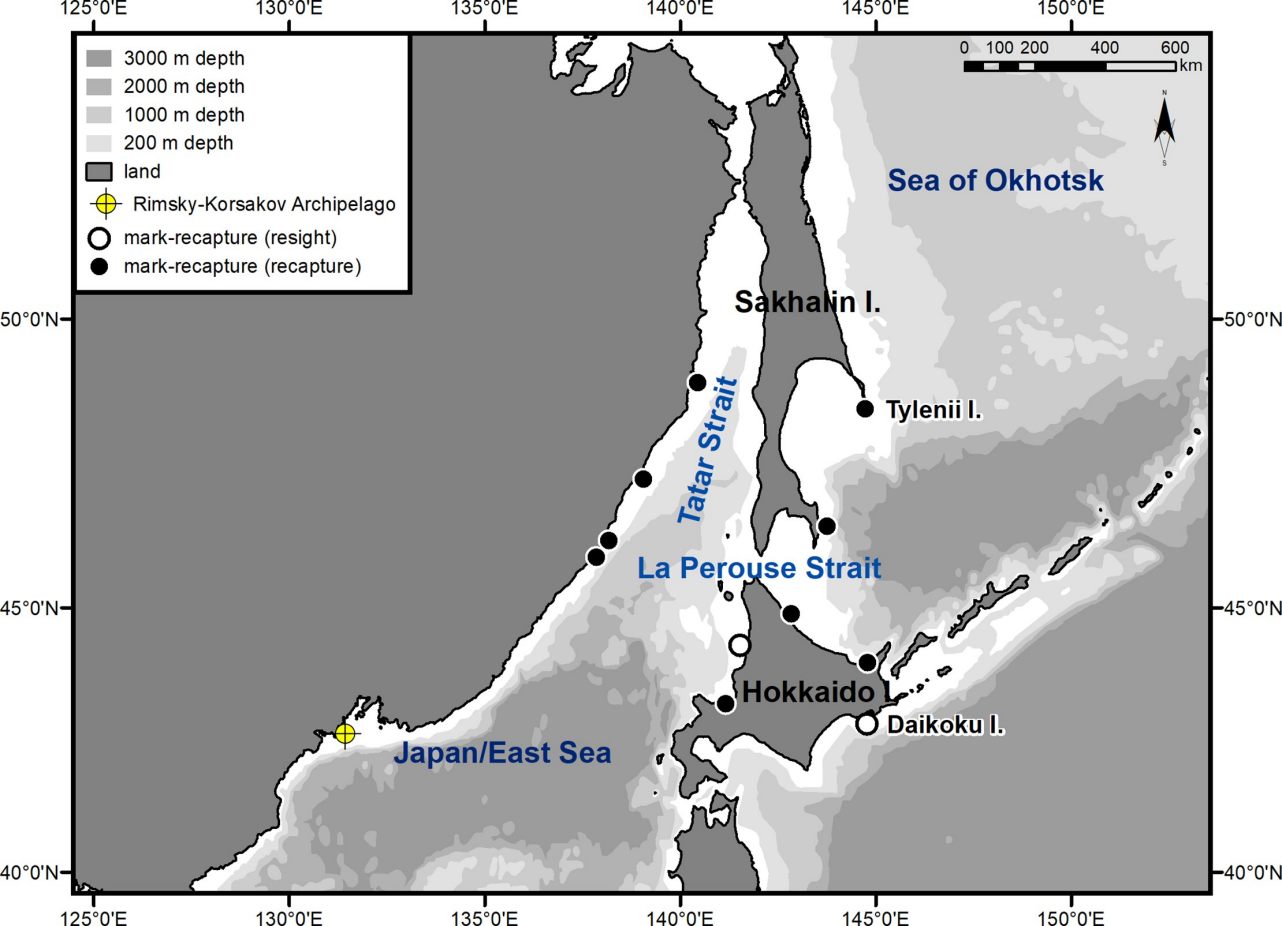
Locations of subsequent findings (recaptures) of spotted seals marked with plastic tags in Peter the Great Bay (according to Trukhin & Mizuno [6], with updates).

The observed decrease in the number of spotted seals in PGB after the completion of the breeding and molting seasons in the local reproductive group, as was recorded in the 1980s, indicate that seals from PGB migrate to their summer and autumn feeding grounds located outside of the Bay [17]. This paper was preceded by diverse studies on spotted seals inhabiting PGB. One of these studies involved marking seal pups with plastic tags. Subsequent findings of some of the tagged young seals outside of PGB provided a general, but rather informative, pattern of long-distance migration made by spotted seals to their summer and autumn feeding grounds (Fig 9).

Young seals went up to 1400 km (by the shortest route from the tagging point to the follow-up point) from PGB and traveled across the Tatar Strait and the southern Sea of Okhotsk to the coasts of Sakhalin and Hokkaido islands in the first year of life. However, plastic tagging of seals did not provide information on such important migration parameters as the timing, direction, and speed of the seals' movements during migration. We elucidated these questions by tagging seals with satellite transmitters.

All three spotted seals left the PGB waters shortly after tagging. Their movements to the feeding areas located in the northwestern part of PGB were usually within coastal waters. As a rule, the seals did not go beyond the 200-m isobath to the pelagic zone. The seals’ main route of migration from the PGB population ran along the coast of the mainland east and north of PGB. This is evidenced by the available data on the number of spotted seals in this coastal area, which increases during the summer and autumn months each year [3, 6, 18, 25].

The seals periodically reduced their daily speed along the migration routes, presumably for feeding or resting. Occasionally, these stopovers coincided with switching of the tags into a "dry" mode, which indicated that during these events the seals could rest at coastal haul-outs. The duration of stopovers was short, and after such events the seals resumed actively moving toward their destination.

The duration of the periods during which tagged seals stayed in relatively limited areas for up to a few months indicates that the seals were feeding there. These key feeding areas were located in different parts of the Sea of Japan/East Sea and varied in size. The relatively small feeding area of the underyearling female was confined to a coastal haul-out site and adjacent waters, whereas the males' feeding areas were more extensive and could be located farther offshore from their haul-outs. An analysis of the food supply of spotted seals in the waters of their summer and autumn habitats confirmed that these key areas were used by the tagged individuals.

The northern Sea of Japan/East Sea, with its high bioproductivity and diversity of species of commercially harvested invertebrates and fish, has long been exposed to increased fishing pressure. The spotted seal is not a benthophage. These seals find food mainly in the pelagiс zone. The juveniles of many bottom-living fish species are mainly pelagic [26] and therefore form part of the diet of spotted seals. A remarkable exception is crustaceans, which are an important part of the diet of young seals. The macrozoobenthos of the northern Sea of Japan/East Sea includes more than 30 species of shrimp alone [27, 28]. In the Tatar Strait, this invertebrate group is dominated by coonstripe shrimp (*Pandalus hypsinotus*), which form dense, commercially harvested aggregations with high biomass [28, 29], and by humpy shrimp (*Pandalus goniurus*), whose density of juveniles at depths of up to 65 m reaches 3.2 g/m^2^ [30]. In addition to these two common species, the pandalid fauna of the Tatar Strait includes several other species that have a lower biomass. Nevertheless, these species are a potential food supply for young spotted seals. Other members of the macrobenthos in the northern Sea of Japan/East Sea are amphipods, which have the highest population densities there [31]. Both shrimp and amphipods are of great importance in the diet of spotted seals during their first year of life [3, 32]. Euphausiids constitute an equally important food item for young spotted seals in the first months of independent life [3, 32, 33]. The euphausiid fauna in the Tatar Strait, consisting of four species [34], is dominated by *Thysanoessa raschii*, which has a reproduction center there and exists in high abundance [35, 36]. Juvenile spotted seals actively feed on euphausiids during the first months after weaning; some stomachs of juvenile seals were found to contain several hundred individuals of these crustaceans [3].

The Tatar Strait and its northwestern shelf have rich ichthyocenoses [37–39] numbering more than a hundred fish species, many of which are frequent items of the spotted seals' diet in different parts of its range. It includes species like greenlings, herring, smelt, and others. But the fish account only for a small proportion of the diet of juvenile spotted seals [3]. Nevertheless, herring is known to have a significant role in the diet of spotted seals everywhere [32, 33, 40, 41]. According to Milovankin [39], in the northern half of the Sea of Japan/East Sea, the largest herring biomass (1.1 t/km^2^) was recorded from the Tatar Strait. There the feeding season of herring [42] overlaps with the summer–autumn feeding period of spotted seals. Many fish species that are actively consumed by spotted seals reach high abundance in the Tatar Strait during the summer and autumn [37, 38, 43–47]. Ichthyocenoses in waters off the western coast of Sakhalin Island also form a significant biomass [48].

In view of the above facts, the Tatar Strait seems to be a region where spotted seals have food available in abundance in the summer and autumn. This especially applies to young seals, for which it is important that the food supply includes a rich fauna of benthic and pelagic invertebrates with high biomasses.

There is enough food for adult spotted seals in the Tatar Strait. There are very dense clusters of Pacific salmon in the Strait. The density of pink salmon there at times can reach the levels observed in the main salmon regions of the Far East [49].

Of particular importance are the coastal haul-outs formed during the summer–autumn feeding period on the western coast of the Tatar Strait, where spotted seals aggregate between their feeding trips [50, 51]. While foraging in the Tatar Strait both underyearling seals regularly showed a significant reduction in horizontal speed around specific sites on the coast of the mainland (hotspot analysis, Gi* bin ≤ – 2). For the underyearling male, this was Chikhacheva Bay and the adjacent Tabo Bay. The underyearling female tended to keep to a limited segment of the coast between capes Peschany and Krasny Partizan. While in these areas, the tags routinely went into a "dry" mode, which indicated the existence of coastal haul-outs. The increased number of spotted seals in the summer and autumn seasons in Chikhacheva Bay was reported by Maminov [51]. This author estimated the number of spotted seals off the western coast of the Tatar Strait in August and September 2008 at 2500 to 3000 individuals. Such a high number of seals in the feeding areas indicates a reliable food supply. It is obvious that the Tatar Strait is a feeding ground not only for spotted seals that come here from PGB, but also for the local reproductive group of seals, which is described as a local population [52].

The tagged yearling seal, after having left the Sea of Japan/East Sea, spent the entire feeding period in Aniva Bay. A study during the 20th century revealed multiple sites in this Bay where spotted seals formed haul-outs of up to several hundred individuals in the summer–autumn period: rocks in Morzha Bay, the estuaries of the Uryum and Taranai rivers, and Aniva Cape [53].

Aniva Bay is relatively shallow; with a maximum depth of 100 to 110 m [54], it is accessible to seals throughout the water column. The Bay is inhabited by an extremely diverse fish fauna during the summer and autumn seasons [26]. The list of fishes recorded from the Bay includes 274 species [55, 56]. Many are common items in the diets of fish-eating animals such as the spotted seal and constitute a significant biomass in the Bay: smelt, capelin, saffron cod, herring, and others. The Bay, with a coastline of up to 230 km [57], receives almost 30 rivers used as spawning grounds by a number of anadromous fish species, such as the pink salmon, which migrates in large numbers across the Bay [58]. Pink salmon constitutes an important food supply for spotted seals in all parts of the Sea of Okhotsk [53, 59, 60]. The yearling male could prey on a variety of fishes that were the main food items in its diet. However, it should also be noted that the macrobenthos and zooplankton communities in Aniva Bay [61, 62] include many other species that are actually and/or potentially consumed by spotted seals.

Thus, the feeding areas of all the tagged spotted seals were characterized by an abundant food supply, with a diverse species composition of food items and high biomass.

Although the feeding areas of the three spotted seals under study were located at a considerable distance from each other, all three seals left their feeding areas and started reverse migrations to the winter habitat (PGB) at approximately the same time, November 3 to 9.

The pattern of the reverse migration varied among the individuals. The underyearling female moved back without delays and entered PGB on November 14. Thus, she covered the entire route within 10 days. Unlike the female, both males made several short stopovers in the initial stage of the reverse migration, during which they probably continued to feed. The underyearling male crossed the eastern boundary of PGB on December 21. The last signal from the yearling male was received on December 11, when the seal was still in waters of the Tatar Strait. On their way back, all the seals completed the migration route in a shorter time than in the spring. The Liman Current undoubtedly has a noticeable effect on the speed of the seals' migration. Flowing from north to south along the coast of the mainland at a velocity of 0.2–0.5 kn (or 9–22 km/day) [63], the current reduces the speed of spotted seals during their northward migration to the summer feeding grounds, but contributes to a faster completion of the reverse migration. In addition, the route of the autumn migration was shorter than the summer route, i.e., in the opposite direction the seals traveled by a straighter route. The higher speed of migration of the yearling seal compared with the underyearling seals may be explained by its better physical condition. Even though we could track the reverse migrations of only two seals, we are inclined to believe that the routes of these migrations were similar to those used by spotted seals moving to their summer feeding grounds in spring.

The seasonal increase in the number of spotted seals in PGB begins annually in late autumn or early winter [3, 19]. Therefore, the time of arrival of the tagged seals fits perfectly into this period. For example, two complete counts of the numbers of spotted seals in the Rimsky-Korsakov Archipelago, carried out on all islands of the archipelago in the autumn of 2014 at weekly intervals, recorded 298 seals on November 20 and 647 seals on November 27 (A.M. Trukhin, unpublished data). It is obvious that such a significant increase in the number of seals within such a short time span is explained by the onset of the mass return of seals to their natal islands after the summer–autumn feeding period that they had spent outside of PGB and completed in the last 10 days of November.

In the early winter, the male and female tagged underyearling seals tended to remain in PGB or Josan Bay. In both areas, they showed the same patterns of movements as those recorded at their summer feeding grounds: the underyearling female fed within a compact key area near the coast (Fig 7, hot area 3), whereas the male made extended feeding trips from the Rimsky-Korsakov Archipelago (Fig 8, rectangle *a*) to remote areas in the pelagic zone of PGB (Fig 8, rectangle *b*). This observation elucidates, in part, where immature seals may stay during the breeding period of the local population. Until recently, the only large aggregation of juvenile and nonbreeding spotted seals in PGB during the breeding season was found at the ice fields in Amur Bay [17].

Prior to the breeding season, both underyearling seals left the waters of the islands and moved from the archipelago. This phenomenon is fully explained by the known pattern of spotted seals' distribution in PGB with differentiation by age and physiological maturity during the breeding season. At this time, the seals' haul-out sites in the Rimsky-Korsakov Archipelago are occupied exclusively by sexually mature animals from the core of the local reproductive group [3, 19].

The first molting aggregations of spotted seals in the Rimsky-Korsakov Archipelago usually occur during the first days of March or, sometimes, a little earlier each year. This event coincides in time with the completion of mass breeding and the simultaneous temporary departure of sexually mature seals from the islands. Adult seals return to the islands by the end of February to join the molting aggregations of immature animals, which also become increasingly more numerous here during this period. The number of molting spotted seals in the Rimsky-Korsakov Archipelago increases during April, with the most pronounced increase occurring in the first half of the month. Thus, in 2016, the dynamics of the number of spotted seals (except for 0-year-old pups) on the islands of the archipelago were as follows: 561 seals were recorded on March 4, 1825 on April 8, 2237 on April 11, and 2737 on April 15 (A.M. Trukhin, unpublished data). The onset of the breeding season coincided with the return of the tagged underyearling male to the haul-out sites in the archipelago. Signals from this male were received until the tag came off along with hair shed during the molt. The underyearling female lost her tag off the coast of the Korean Peninsula.

It should be noted that not all seals exhibit such migration behavior. Each year, a small part of the population does not go beyond the boundaries of PGB, leading a relatively sedentary life here, or leaves the bay for a short distance and for a short time [3, 19]. Spotted seals' migrations outside PGB are undoubtedly determined by trophic factors and can be considered an actual strategy of the population that allows more complete use of food resources available within the range.

All researchers who previously tagged spotted seals with satellite transmitters [4–9], caught animals not at their breeding sites, but much later, during the summer feeding period. Therefore, it was sometimes not known to which reproductive group the tagged individuals belonged. We caught and tagged young spotted seals at the breeding grounds, which made it possible, for the first time, to collect information on their migration and the pattern of use of seasonal habitats of this species throughout the entire annual cycle.

## Conclusion

Feeding migrations of young spotted seals born in Peter the Great Bay begin immediately after the completion of the molt that occurs at the coastal haul-out sites on the islands of the Rimsky-Korsakov Archipelago. The migration routes of different individuals to and from their summer feeding grounds are similar and run along the coast of the mainland. The feeding areas of young spotted seals are limited in size, and seals stay within their boundaries throughout the summer–autumn feeding period (until November). In addition to the main key area, there may be one or more additional areas at the summer and autumn grounds where animals either feed or rest at coastal haul-outs. All feeding areas are characterized by abundant food supplies. In the Tatar Strait, juvenile seals are believed to feed mainly on common shrimp species of the family Pandalidae and on other crustacean species, which form dense aggregations with high biomasses in this region, as well as, to a lesser extent, on fish. The food supply for spotted seals in Aniva Bay can be the local fish fauna, including a variety of species. After completing their feeding migrations and returning to their natal habitats in the Rimsky-Korsakov Archipelago, seals form large aggregations on the islands in December and early January. However, by the onset of the breeding season (January), only the sexually mature part of the population remains there, while immature seals move to a significant distance off the coast of the archipelago, including the pelagic zone. Their return to the haul-out sites is associated with the onset of molt and coincides with completion of the breeding season.

## Supporting information

S1 File Normality tests of distribution (Shapiro-Wilk normality test) in sets of daily speeds of migrating spotted seals. (XLSX)

S2 File Analysis of Variance (ANOVA) between sets of daily speeds of migrating spotted seals. (XLSX)

S3 File Testing for difference (unpaired two-tailed pairwise t-tests) between sets of daily speeds of migrating spotted seals. (XLSX)
