# Correction: Migrations of young spotted seals (*Phoca largha*) from Peter the Great Bay, Sea of Japan/East Sea, and the pattern of their use of seasonal habitats

**DOI:** 10.1371/journal.pone.0257121

**Published:** 2021-09-01

**Authors:** Alexey M. Trukhin, Peter A. Permyakov, Sergey D. Ryazanov, Vyacheslav B. Lobanov, Hyun Woo Kim, Young Min Choi, Hawsun Sohn

Fig 8 is incorrect. The authors have provided a corrected version here.

**Fig 8:**
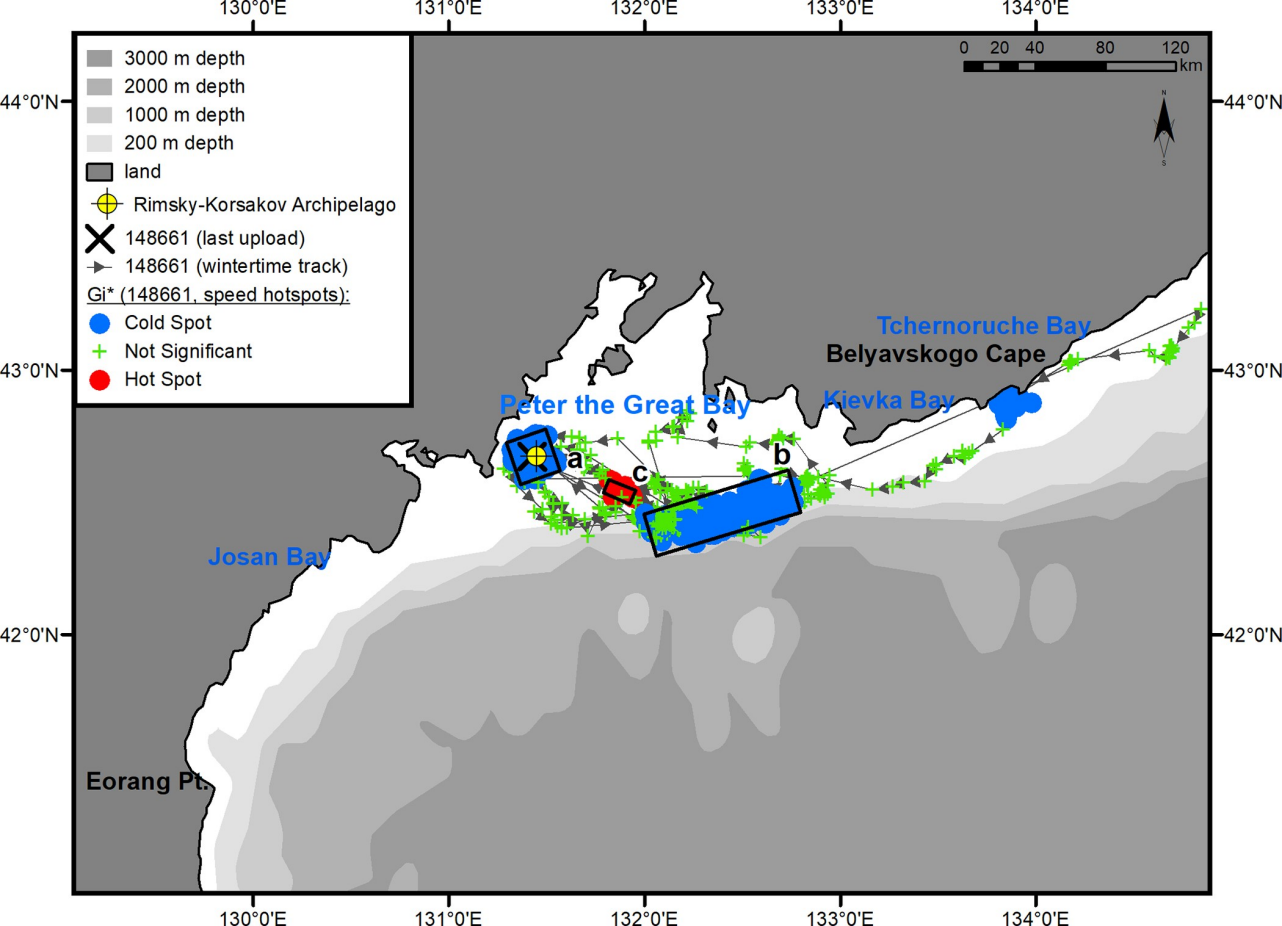
Winter habitats of underyearling male #148661. The numbers of the hot areas are the same as in Fig 3.
